# Supplementary material for: Short-Term CO2 Treatment of Harvested Grapes (Vitis vinifera L., cv. Trebbiano) before Partial Dehydration Affects Berry Secondary Metabolism and the Aromatic Profile of the Resulting Wine
Source: Plants (Basel). 2022 Jul 29;11(15):1973. doi: 10.3390/plants11151973 (PMC9370517; doi:10.3390/plants11151973)
Supplement: Supplementary file 1 [file plants-11-01973-s001.zip › plants-1819537-supplementary.pdf]

Short-term CO_2 treatment of harvested grapes (*Vitis vinifera* L., cv. Trebbiano) before partial dehydration affects berry secondary metabolism and the aromatic profile of the resulting wine

Marco Santin [1§], Stefano Brizzolara [2§], Antonella Castagna [1], Annamaria Ranieri [1,*] and Pietro Tonutti [2]

[1] Department of Agricultural, Food and Agro-Environmental Sciences (DAFE), University of Pisa, Via del Borghetto 80, 56100 Pisa, Italy; marco.santin@unipi.it; antonella.castagna@unipi.it; anna.maria.ranieri@unipi.it
[2] Crop Science Research Center, Scuola Superiore Sant'Anna, Piazza Martiri della Libertà 33, 56127, Pisa, Italy; stefano.brizzolara@santannapisa.it; pietro.tonutti@santannapisa.it
* Correspondence: anna.maria.ranieri@unipi.it;
§ These authors have contributed equally to this work and share first authorship

Table S1 - Carotenoids, chlorophylls e xanthophylls in Trebbiano grape skin during postharvest storage inside the dehydration chamber

	T0	T1		T2	
		Control	Treated	Control	Treated
**Beta-carotene**	1.91 ± 0.16 a	**1.28 ± 0.05 b**	**2.293 ± 0.09 a**	1.02 ± 0.02 b	1.285 ± 0.098 b
**Lutein**	0.51 ± 0.14 a	0.67 ± 0.39 a	0.69 ± 0.05 a	0.49 ± 0.006 a	0.48 ± 0.01 a
**Zeaxanthin**	0.42 ± 0.024 a	0.065 ± 0.037 b	0.076 ± 0.0098 b	0.058 ± 0.0075 b	0.054 ± 0.02 b
**Neoxanthin**	0.14 ± 0.054 a	0.037 ± 0.009 b	0.045 ± 0.0037 b	0.03 ± 0.0028 b	0.028 ± 0.0023 b
**Violaxanthin**	0.15 ± 0.02 a	**0.078 ± 0.013 b**	**0.153 ± 0.019 a**	0.069 ± 0.0094 b	0.036 ± 0.0306 b
**Anteraxanthin**	0.24 ± 0.015 a	0.017 ± 0.0065 b	0.013 ± 0.014 b	0.022 ± 0.0078 b	0.0056 ± 0.0055 b
**Chlorophyll a**	8.06 ± 0.59 ab	**5.747 ± 0.136 bc**	**9.813 ± 0.086 a**	3.806 ± 0.131 c	5.678 ± 0.294 c
**Chlorophyll b**	1.74 ± 0.13 ab	**1.23 ± 0.02 c**	**2.09 ± 0.14 a**	**0.79 ± 0.05 d**	**1.34 ± 0.07 c**

Table S1 - Carotenoids, chlorophylls and xanthophylls content (µg/g fresh weight) in Trebbiano berry skin of control and treated berries allowed to wither inside a dehydration chamber, at harvest time (T0), at 30% weight loss (T1), and at the end of the drying period (T2) when berries lost about 50% of weight. Treated berries were incubated for 24 h with CO_2 (30%, 14° C), and then allowed to wither at this temperature, while control berries were not subjected to the treatment and allowed to wither at the same environmental parameters. Statistically significant differences were evaluated by one-way ANOVA ($p \leq 0.05$), and different letters indicate significantly different values according to Tukey–Kramer test.

Table S2 - Phenolic classes in Trebbiano grape skin (2008) during post-harvest storage inside the dehydration chamber

	T0	T1		T2	
		Control	Treated	Control	Treated
**Total phenols (mg GAE/g d.w.)**	9.99±0.	14.47±1.84 a	11.33±0.64 ab	9.9±2.63 b	7.99±0.516 b
**Flavonoids (mg CTCE/g d.w.)**	6.99±0.	**11.56±0.17 a**	**8.64±0.47 b**	**5.89±0.43 c**	**3.71±1.076 d**
**Flavonols (mg QCTE/g d.w.)**	0.56±0.	0.83±0.049 a	0.64±0.005 ab	0.22±0.07 c	0.41±0.069 bc
**Proanthocyanidins (mg CYAE/g d.w.)**	0.99±0.	1.74±0.17 a	1.51±0.11 a	1.18±0.075 ab	0.70±0.12 b
**Flavan-3-ols (mg CTCE/g d.w.)**	3.87±0.	5.45±0.044 a	4.19±0.37 a	2.69±0.13 ab	1.56±0.37 b
**Tartaric acid esters (mg CAE/g d.w.)**	0.51±0.	0.92±0.1 a	0.61±0.067 a	0.32±0.0024 a	0.40±0.082 a

Table S2 - Phenol content in Trebbiano berry skin of control and treated berries allowed to wither inside a dehydration chamber, at harvest time (T0), at 30% weight loss (T1), and at the end of the drying period (T2) when berries lost about 50% of weight. Treated berries were incubated for 24 h with CO_2 (30%, 14° C), and then allowed to wither at this temperature, while control berries were not subjected to the treatment and allowed to wither at the same environmental parameters. Statistically significant differences were evaluated by one-way ANOVA ($p \leq 0.05$) and different letters indicate significantly different values according to Tukey–Kramer test. GAE: gallic acid equivalents; CTCE: catechin equivalents; QCTE: quercetin equivalents; CYAE: cyanidin equivalents; CAE: caffeic acid equivalents; d.w.: dry weight.

Table - S3 Free aroma compounds in Trebbiano wine (µg/L)					
**Aromatic families**		**Control**	**Treated**	**Odor threshold (µg/L)**	**Reference**
**Acids**	**Propanoic acid**	176.57 ± 28.5	192.65 ± 58.5	20000	[1]
	**Isovaleric acid + 2-methylbutyric acid**	554.72 ± 1.3	717.91 ± 127.1	700*	[1]
	**Hexanoic acid (caproic acid)**	1223.52 ± 61.3	1132.02 ± 41.1	1000-10000, 8800	[1,2]
	**Octanoic acid (caprylic acid)**	1167.00 ± 517.7	977.33 ± 153.9	15000	[1]
	**Decanoic acid (capric acid)**	**963.99 ± 102.5 a**	**72.97 ± 10.2 b**	8200	[1]
	**Hexadecanoic acid (palmitic acid)**	**552.43 ± 76.4**	**n.d.**	**-**	
	**Isobutyric acid**	401.73 ± 290.7	694.01 ± 79.5	8100	[1]
**Dioxanes and dioxolanes**	**N-ethylacetamide**	118.74 ± 8.9	198.61 ± 117.5	-	
**Alcohols**	**1-propanol**	7183.62 ± 796.3	5784.30 ± 2010.5	-	
	**Isobutanol**	7950.99 ± 4625.4	8893.60 ± 1349.8	75000 40000	[2,3]
	**1-butanol**	377.03 ± 98.8	309.00 ± 50.2	40000	[4]
	**Isoamylic alcohol**	75094.42 ± 53935.9	87152.11 ± 3223.0	7000	[1]
	**3-methyl-1-pentanol**	105.20 ± 23.0	74.50 ± 30.4	-	
	**3-ethoxy-1-propanol**	1320.42 ± 311.4	1998.30 ± 348.3	-	
	**2,3-butanediol**	**3318.60 ± 514.6 b**	**32459.2 ± 2956.9 a**	40000	[5]
	**1,2-propanediol**	**444.28 ± 163.3 b**	**1207.75 ± 507.3 a**	**-**	
	**2-furanmethanol**	1791.57 ± 212.1	1534.49 ± 208.9	-	
	**2-phenylethanol**	17433.69 ± 3063.0	20937.82 ± 426.8	7500	[1]
	**Glycerol**	533.43 ± 202.7	677.20 ± 180.2	-	
	**Tyrosol**	1505.55 ± 724.8	2406.2 ± 303.3	-	
**Esters**	**Isoamyl acetate**	316.48 ± 92.6	308.80 ± 10.2	200	[1]
	**Ethyl hexanoate**	437.40 ± 62.2	415.20 ± 293.6	65, 14, 5	[3,6,7]
	**Ethyl lactate**	256.46± 50.7	236.50 ± 32.8	14000	[1]
	**Acetic acid+ethyl octanoate**	23608.34 ± 12429.6	24100.30 ± 11186.6	250**	[1]
	**Ethyl-3-hydroxy butyrate**	103.00 ± 72.8	313.57 ± 163.1	-	
	**Diethyl succinate**	**374.60 ± 2.3 b**	**463.42 ± 36.2 a**	100000	[5]
	**Ethyl-9-decanoate**	**27.70 ± 19.6**	**n. d.**	200	[6]
	**1,3 propanediol monoacetate**	**459.82 ± 43.2 b**	**894.12 ± 315.4 a**	**-**	
	**Ethyl 4-hydroxybutanoate**	13458.93 ± 1015.5	17427.80 ± 4017.1	-	
	**Ethyl decanoate**	**341.71 ± 81.0 a**	**171.58 ± 58.3 b**	200	[6]
	**Diethyl malate**	**58.40 ± 41.3**	**n. d.**	760000	[5]
	**4-hydroxy-isopenthyl butanoate**	185.59 ± 26.5	260.68 ± 65.4	-	
	**Ethyl c16**	**988.82 ± 601.7 a**	**134.40 ± 95.0 b**	-	
	**Ethyl-γ-hexadecanoate**	**178.39 ± 89.3 a**	**42.20 ± 29.8 b**	-	
	**Mono ethyl succinate**	5010.88 ± 1302.1	4150.00 ± 1792.5	1000000	[5]
	**Ethyl c18**	**334.58 ± 37.4**	**n. d.**	-	
	**Ethyl oleate**	**303.17 ± 24.4**	**n. d.**	-	
	**Ethyl linoleate**	**218.77 ± 178.7**	**n. d.**	-	
**Volatile phenols**	**4-vinyl guaiacol**	126.03 ± 8.2	217.14 ± 127.9	10, 40	[7,8]
	**Vanillin**	**16.88 ± 3.9**	**n. d.**	200	[3]
	**Methyl vanillate**	**4.36 ± 2.2**	**n. d.**	-	
	**Ethyl vanillate**	**53.75 ± 6.0**	**n. d.**	-	
	**Acetovanillone**	**150.35 ± 43.0**	**n. d.**	-	
	**Zingerone (vanillylacetone)**	**13.79 ± 9.5**	**n. d.**	-	
	**Guaiacyl propanol**	**351.64 ± 164.7**	**n. d.**	-	
	**Guaiacyl ethanol**	**295.35 ± 213.2**	**n. d.**	-	
**Other molecules**	**Acetoin**	1694.85 ± 618.2	2029.29 ± 1036.1	150000	[7]
	**1-hexanal**	529.87 ± 90.9	486.72 ± 3.2	5	[9]
	**Furfural**	2331.91 ± 490.6	1308.34 ± 543.8	770	[5]
	**γ-butyrolactone + acid c4**	**1663.11 ± 148.6 a**	**1201.35 ± 217.0 b**	1000***	[5]
	**Methionol**	**75.73 ± 9.3 a**	**25.48 ± 36.0 b**	1000	[7]
	**2-hydroxy-4-piranone**	249.50 ± 57.7	1021.1 ± 932.9	-	
	**Pentalactone**	**24.43 ± 4.5**	**n. d.**	-	
	**4-carbethoxy-γ-butyrolactone precursor**	**62.34 ± 21.6 a**	**24.81 ± 5.1 b**	-	
	**4-carbethoxy-γ-butyrolactone**	18.17 ± 11.7	6.95 ± 2.8	-	
**26.30 ± 7.2**	**3-oxo-α-ionol**	**26.30 ± 7.2**	**n. d.**	-	

* The threshold value is referred only to isovaleric acid.

**The threshold value is referred only to ethyl octanoate.

***The threshold value is referred only to γ-butyrolactone.

Table S3 - Free aroma compound content in Trebbiano wine (µg/L) obtained from totally air-stored berries (control) and CO_2-treated berries (treated), together with the odor threshold values (µg/L) found in the literature. Control and treated berries were allowed to wither inside a dehydration chamber. Treated berries were incubated for 24 h with CO_2 (30%, 14° C), and then allowed to wither at this temperature, while control berries were not subjected to the treatment and allowed to wither at the same environmental parameters. Different letters indicate statistically significant differences according to t-test ($p \leq 0.05$). Means not followed by any letters are not statistically different (t-test, $p \leq 0.05$).

Table S4 - Glycosylated bound aroma compounds in Trebbiano wine (µg/L)					
**Aromatic families**		**Control**	**Treated**	**Odor threshold (µg/L)**	**Reference**
**Alcohols**	**Hexanol**	**5.40 ± 3.0 b**	**13.19 ± 2.3 a**	2500	[5]
	**Octanol**	11.76 ± 1.9	11.41 ± 1.5	-	
	**Myrtenol**	2.11 ± 0.3	2.31 ± 0.2	-	
	**2-(2-butoxyethoxy)-ethanol**	6.88 ± 0.7	7.94 ± 1.1	-	
	**Benzyl alcohol**	8.82 ± 0.1	8.67 ± 0.2	-	
	**2-phenyl ethanol**	10.84 ± 0.8	10.72 ± 0.6	-	
**Volatile phenols**	**Eugenol**	1.71 ± 0.1	2.04 ± 0.3	6, 5	[3,7]
	**Methyl vanillate**	7.48 ± 1.1	6.60 ± 1.2	-	
	**Acetovanillone**	7.77 ± 0.7	8.40 ± 1.3	-	
	**Zingerone (vanillylacetone)**	12.88 ± 1.5	15.43 ± 2.6	-	
**Terpenes**	**linalool oxide furanoid (LOF) CIS**	**n. d.**	**0.47 ± 0.1**	6000	[5]
	**LOF TRANS**	**n. d.**	**0.63 ± 0.2**	6000	[5]
	**Linalool**	**3.02 ± 1.4 b**	**6.40 ± 0.4 a**	50	[5]
	**α-terpineol**	**7.04 ± 0.8 b**	**12.50 ± 1.3 a**	250	[7]
	**LOP (linalool oxide pyranic) TRANS**	0.69 ± 0.3	0.73 ± 1.0	-	
	**LOP CIS+ methyl salicylate**	1.09 ± 0.4	1.40 ± 0.6	-	
	**3,7-diol**	1.03 ± 0.2	1.00 ± 0.0	-	
	***p*-cymen-α-ol**	3.06 ± 0.2	3.57 ± 0.9	-	
	***p*-1-menthen-7,8-diol**	10.02 ± 0.6	9.82 ± 0.1	-	
	**Citronellol**	5.69 ± 0.9	8.10 ± 0.6	100	[3]
	**Nerol**	28.90 ± 0.8	27.20 ± 1.5	60	[10]
	**Geraniol**	148.10 ± 15.6	171.95 ± 35.0	30	[3]
	**Exo-2-hydroxycineole**	10.84 ± 1.0	10.81 ± 1.5	-	
	**Linalool hydrate**	0.92 ± 0.1	1.03 ± 0.3	-	
	**Citronellol hydrate**	**17.37 ± 0.5 b**	**24.40 ± 2.7 a**	-	
	**3,8-diol + nerol hydrate**	**n. d.**	**7.60 ± 0.9**	-	
	**(Z)-8-hydroxylinalool + geraniol hydrate**	**14.93 ± 0.0 b**	**21.02 ± 1.3 a**	-	
**Esters**	**Ethyl caprate**	0.94 ± 0.1	1.20 ± 0.3	-	
	**Isopropyl myristate**	9.91 ± 1.1	9.80 ± 1.9	-	
**C13**	**3,4-dihydro-3-oxo-actinidol I (ACTI I)**	4.37 ± 0.3	5.65 ± 1.0	-	
	**ACTI II**	5.52 ± 0.5	5.60 ± 1.0	-	
	**ACTI III**	6.92 ± 0.3	7.68 ± 1.0	-	
	**ACTI IIII + 3 hydroxy-β-damascone**	53.71 ± 0.5	56.48 ± 10.2	-	
	**3-hydroxy-7,8-dihydro-β-ionone**	9.90 ± 0.5	11.31 ± 1.9		
	**3-oxo-α-ionol + 4-oxo-β-ionol**	118.64 ± 7.8	133.29 ± 24.9	-	
	**3-hydroxy-7,8-dihydro-β-ionol**	**55.30 ± 6.1 b**	**100,20 ± 11.7 a**	-	
	**4-oxo-7,8-dihydro-β-ionol + hydroxy-β-ionone**	20.76 ± 2.6	19.51 ± 3.0	-	
	**3-oxo-7,8-dihydro-α-ionol**	**6.72 ± 0.3 b**	**9.35 ± 1.6 a**	-	
	**3-oxo-retro-α-ionol**	6.10 ± 0.5	6.66 ± 0.3	-	
	**7,8-dihydrovomifoliol**	23.69 ± 3.4	18.73 ± 5.8	-	
	**Vomifoliol**	4.90 ± 1.2	4.15 ± 2.1	-	

Table S4 - Glycosilated-bound aroma compound content in Trebbiano wine (µg/L) obtained from totally air-stored berries (control) and CO_2-treated berries (treated), together with the odor threshold values (µg/L) found in the literature. Control and treated berries were allowed to wither inside a dehydration chamber. Treated berries were incubated for 24 h with CO_2 (30%, 14° C), and then allowed to wither at this temperature, while control berries were not subjected to the treatment and allowed to wither at the same environmental parameters. Different letters indicate statistically significant differences according to t-test ($p \leq 0.05$). Means not followed by any letters are not statistically different (t-test, $p \leq 0.05$).
